# Evaluation of Antimicrobial Efficacy of Arjuna, Bromelain, Calcium Hydroxide, and Triple Antibiotic Paste as an Intracanal Medicament Against Enterococcus faecalis: An In Vitro Study

**DOI:** 10.7759/cureus.96968

**Published:** 2025-11-16

**Authors:** Ranjana H Deshmukh, Neelam Chandwani, Anjali Gayke, Neeta Gade, Reetika Reetika, Manish Shrigiriwar

**Affiliations:** 1 Conservative Dentistry and Endodontics, All India Institute of Medical Sciences, Nagpur, Nagpur, IND; 2 Dentistry, All India Institute of Medical Sciences, Nagpur, Nagpur, IND; 3 Microbiology, All India Institute of Medical Sciences, Nagpur, Nagpur, IND; 4 Forensic Medicine, All India Institute of Medical Sciences, Nagpur, Nagpur, IND

**Keywords:** anti-bacterial agents, antimicrobial efficacy, bromelain, calcium hydroxide, endodontics, enterococcus faecalis, terminalia arjuna, triple antibiotic paste

## Abstract

Introduction: *Enterococcus faecalis* is a common pathogen associated with endodontic treatment failure due to its resistance to conventional therapies.

Aim: This study aimed to evaluate and compare the antimicrobial efficacy of two novel herbal agents, *Terminalia arjuna* (Arjuna) and bromelain, against calcium hydroxide (CaOH) and triple antibiotic paste (TAP) for their potency in eliminating *Enterococcus faecalis*.

Methods: Eighty human teeth with single roots were endodontically prepared and divided into five groups: Group 1 (Arjuna paste), Group 2 (CaOH), Group 3 (TAP), Group 4 (bromelain paste), and Group 5 as a control with no medicament. After bacterial inoculation and a three-week incubation period, canals were instrumented and irrigated. Antimicrobial efficacy was assessed by measuring the reduction in bacterial colony-forming units (CFUs) after seven days of intracanal medicament application.

Results: The highest percentage of bacterial reduction was seen in TAP (99.89%), followed by bromelain paste (98.57%) and Arjuna paste (99.02%). CaOH showed the lowest reduction (93.27%) in bacterial load.

Conclusion: The results suggest that while TAP remains a highly effective intracanal medicament against *E. faecalis*, *Terminalia arjuna* and bromelain exhibited considerable potential as a natural alternative with antimicrobial properties, while calcium hydroxide showed the least effectiveness.

## Introduction

The long-term success of endodontic therapy is predicted on the thorough elimination of bacterial contamination from the root canal (RC) system. RC treatment failure is a multifactorial issue that primarily arises due to persistent microbial infection, particularly within one-third of the canal apically. *Enterococcus faecalis* (EF), a gram-positive, facultative anaerobic bacterium commonly found in the human oral cavity, gastrointestinal tract, and genitourinary tract, has been notably implicated in approximately 38% of RC failure cases [1]. This is attributable to its remarkable resilience in harsh environments, including nutrient-poor environments, high alkaline pH, and its ability to penetrate dentinal tubules, providing protection from intracanal medicaments [2].

The bacterium’s virulence factors, including extracellular proteases and cytolysin, contribute to tissue destruction and inflammation in periapical tissues. Due to these characteristics, EF remains one of the most studied microorganisms in endodontics related to RC treatment failure [3]. Commonly, calcium hydroxide (CH), a very traditional and yet still used intracanal medicament, is widely employed due to its high alkalinity. This weakens the bacterial cell walls and denatures essential proteins [4]. However, several studies have demonstrated that CH exhibits limited efficacy against EF, which can persist within dentinal tubules despite treatment [5].

In recent decades, triple antibiotic paste (TAP), a mixture of three antibiotics (metronidazole, ciprofloxacin, and minocycline), has been widely considered as an alternative medicament, showing efficacy against a broad range of endodontic pathogens. Nevertheless, TAP is associated with disadvantages such as tooth discoloration, primarily due to minocycline [6], the potential for promoting antibiotic resistance, hypersensitivity reactions, and concerns regarding tissue biocompatibility [7].

In recent years, to overcome these limitations, novel medications, particularly those derived from phytochemicals, have been investigated. Bromelain and *Terminalia arjuna* are two of such widely investigated phytochemicals in endodontics [8,9]. Bromelain, a proteolytic enzyme complex derived from pineapple (*Ananas comosus*), has been studied primarily for its anticariogenic effects, antimicrobial effects in endodontics, and anti-inflammatory regimen in post-extraction cases [8].

*Terminalia arjuna*, also known as "Arjuna," is a plant with medicinal properties that is native to India. It has various medicinal properties, including antidyslipidemic, hypocholesterolemic, antioxidant, anticancer, and antibacterial activities [9,10].

There are currently no comparative microbiological investigations evaluating the antibacterial effectiveness of TAP, CH, *T. arjuna*, and bromelain as intracanal medications against EF. Therefore, the purpose of this study was to assess and compare the antibacterial activity of two new herbal agents, Arjuna and bromelain, with the most widely used intracanal medications, TAP and CaOH, against EF.

## Materials and methods

This in vitro trial was conducted in the Department of Dentistry between March 2025 and June 2025. Institutional Ethical Committee approval was obtained for this in vitro study (IEC/Pharmac/2025/1213). The study sample included 80 single-rooted human teeth extracted between January 2025 and March 2025.

Sample size calculation

The sample size was calculated based on the data from the study by Chandwani ND and colleagues [11], and the predicted sample size (n) was 80 (i.e., 16 samples per group) [11,12].

Selection criteria

Human teeth that were with single roots and extracted for either periodontal conditions or for orthodontic reasons were chosen. Teeth with resorptions, root caries, RC calcifications, or bent roots were not included. Radiography was used to validate each criterion.

Study procedure

1. Sample collection: Freshly extracted single-rooted teeth were included in the study.

2. Tooth preparation: The soft tissues, calculus, and bone on the root surfaces of 80 included teeth were softly scraped with a periodontal curette. The teeth were soaked for 1 hour in 5.25% NaOCl (Prime Dental Products Pvt. Ltd, Mumbai, India) and then they were preserved in 0.9% physiological saline. The roots were standardized to 16 mm length, while the crowns of the sample teeth were cut at the level of the cementoenamel junction perpendicularly to the tooth’s long axis.

**Figure 1:**
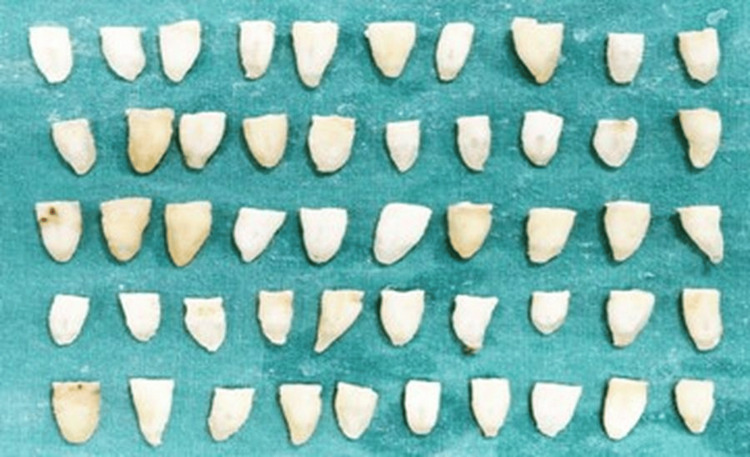
Samples prepared in the present study

Access cavities were prepared with round and Endo-Z burs and K-type file (Mani Inc., Tochigi, Japan) (10/15 size), and the working length was found. Again, the canal was instrumented up to size 25 of K-file with intermittent irrigation with distilled water. Composite was then used to close the foramen. The samples were divided into five groups: Grp1: Arjuna; Grp2: CH; Grp3: TAP; Grp4: bromelain; Grp5: without medicament (Figure 1).

3. Biofilm development and canal inoculation: EF (ATCC 29212) was propagated using sterilized brain heart infusion broth (BHI) (Himedia Laboratories Pvt. Ltd., Mumbai, India), which was cultured in an anaerobic environment for 24 hours at 37°C. To achieve the #0.5 McFarland turbidity level, the bacterial concentration was increased to 1.5 x 10⁸ CFU/ml. Using a 1 ml insulin syringe, 0.5 ml of the suspension was injected into each specimen. The working length of the RC was reached by moving a size #15 K-file up and down. After that, the RC orifices were sealed with a tin foil to avoid leakage. They were then collected into sterile tubes and kept vertically on perforated trays for three weeks at 37°C. The medium was changed once every three days. After incubation, residual broth was removed using paper points, and the initial microbial sample (S1) was collected.

4. RC preparation and medicament application: RC was prepared in all teeth to a size #50 file in a step-back pattern. Ethylenediaminetetraacetic acid (EDTA) (Prime Dental Products Pvt. Ltd, Mumbai, India) and a 2.5% sodium hypochlorite (NaOCl) solution were used for irrigation (17%). The prepared medications were then inserted into the RCs using a lentulo spiral, and the samples were divided into five groups: group 1 received Arjuna, group 2 received CH, group 3 received TAP, group 4 received bromelain, and group 5 received no intracanal medication.

5. Preparation of intracanal medicaments

● Group 1: To create a paste-like consistency, distilled water and Arjuna powder (Shree Botanical Resources, Ahilyanagar, India) were combined in a 1:1 ratio.

● Group 2: To achieve a paste-like consistency, CH powder (Calcicur, VOCO GmBh, Cuxhaven, Germany) was combined with distilled water at a weight/volume ratio of 1.5:1.

● Group 3: Metronidazole (Metrogyl, Unique Pharmaceutical Laboratories, Mumbai, India), minocycline (Minocin, Lederle Laboratories, Pearl River, NY, USA), and ciprofloxacin (Ciplox, Cipla Ltd., Mumbai, India) make up TAP. Each tablet's contents were crushed with a mortar and pestle and combined in a mixing pad in equal weight ratios (1:1) (100 mg each). To create 1 mg/ml of TAP with a creamy consistency, they were all combined and dissolved in 100 ml of sterile water.

● Group 4: bromelain paste - saline was combined in a 1:1 ratio with bromelain powder (Krishna Enzytech Pvt Ltd, Yeola, India), which has an enzyme activity of 2400 gelatin-digesting units per gram. One milliliter of distilled water was combined with 1 g of powder to obtain a creamy paste.

● Group 5 (control group): canals will be left without any medication.

Study outcomes

**Figure 2:**
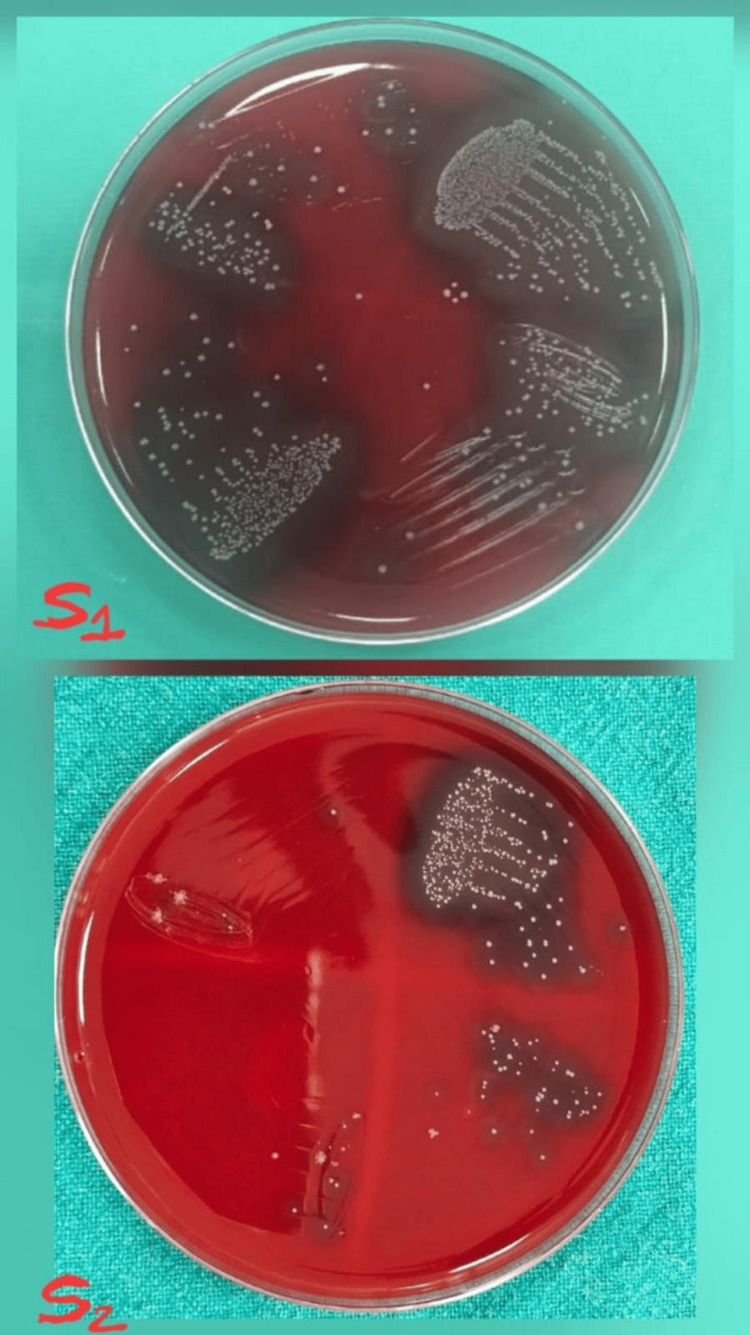
Culture images of the microbial S1 and S2 samples treated with Arjuna S1: initial microbial sample; S2: second microbial sample obtained after seven days of medicament application.

**Figure 3:**
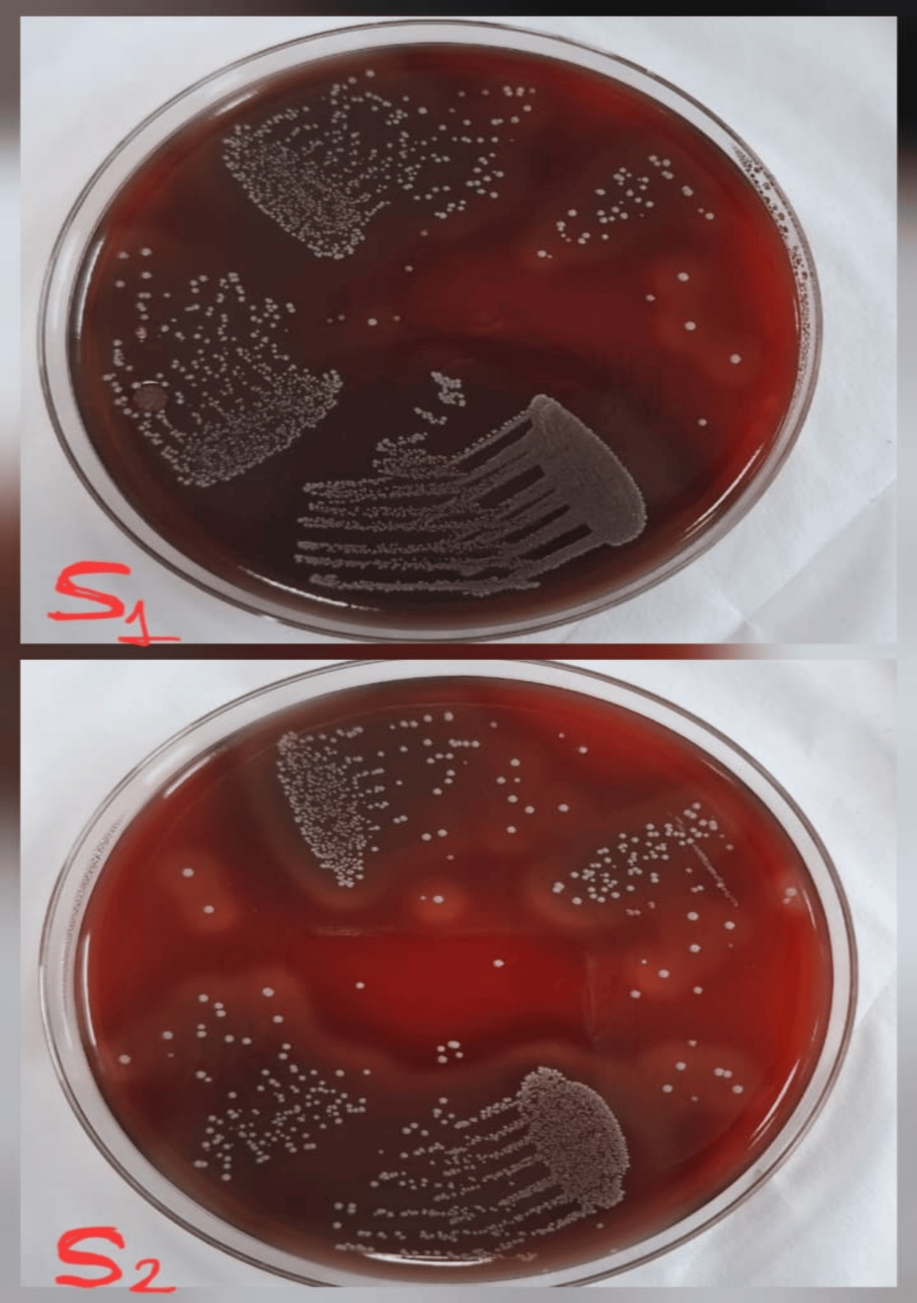
Culture images of the microbial S1 and S2 samples treated with calcium hydroxide S1: initial microbial sample; S2: second microbial sample obtained after seven days of medicament application.

**Figure 4:**
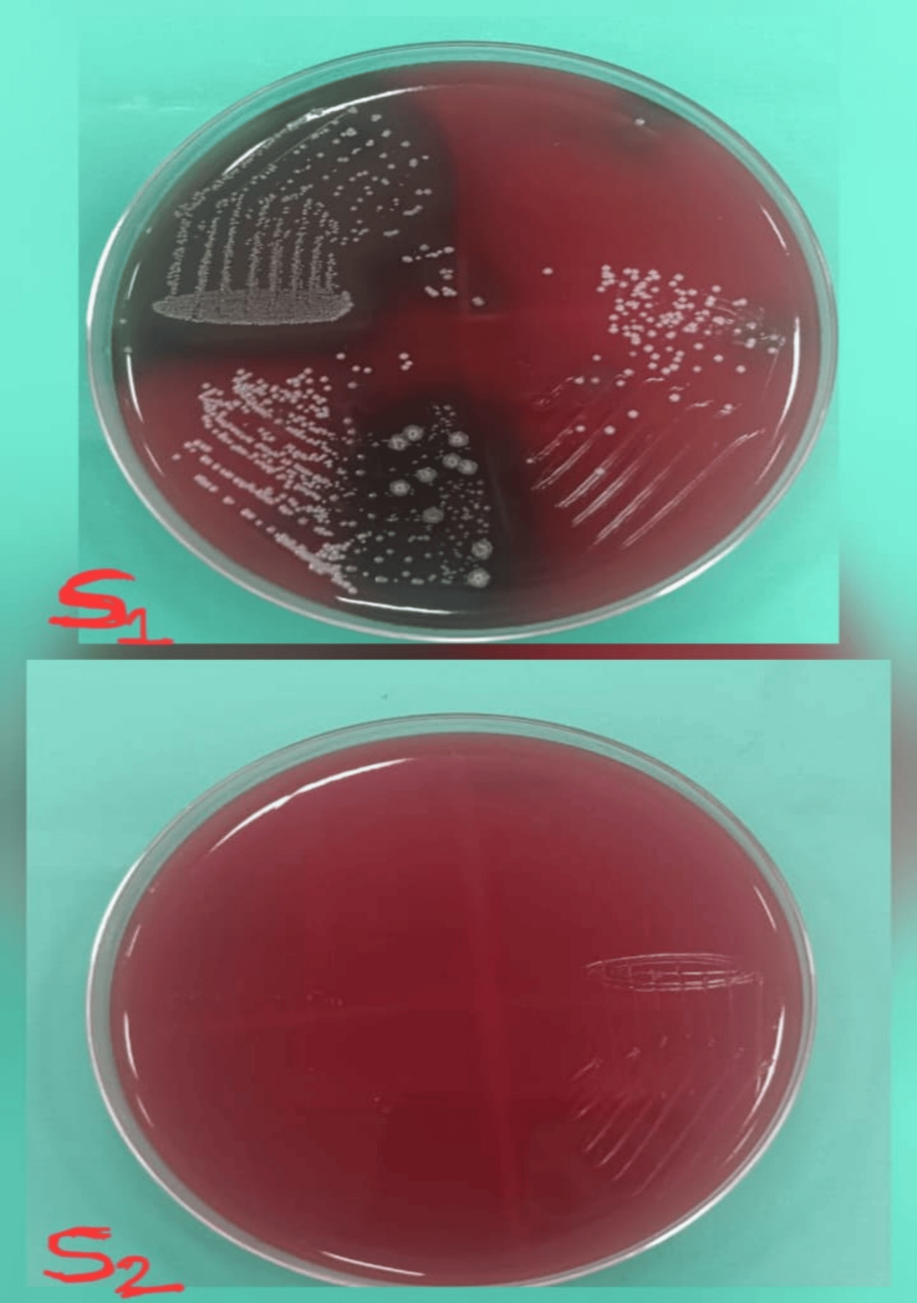
Culture images of the microbial S1 and S2 samples treated with triple antibiotic paste S1: initial microbial sample; S2: second microbial sample obtained after seven days of medicament application.

**Figure 5:**
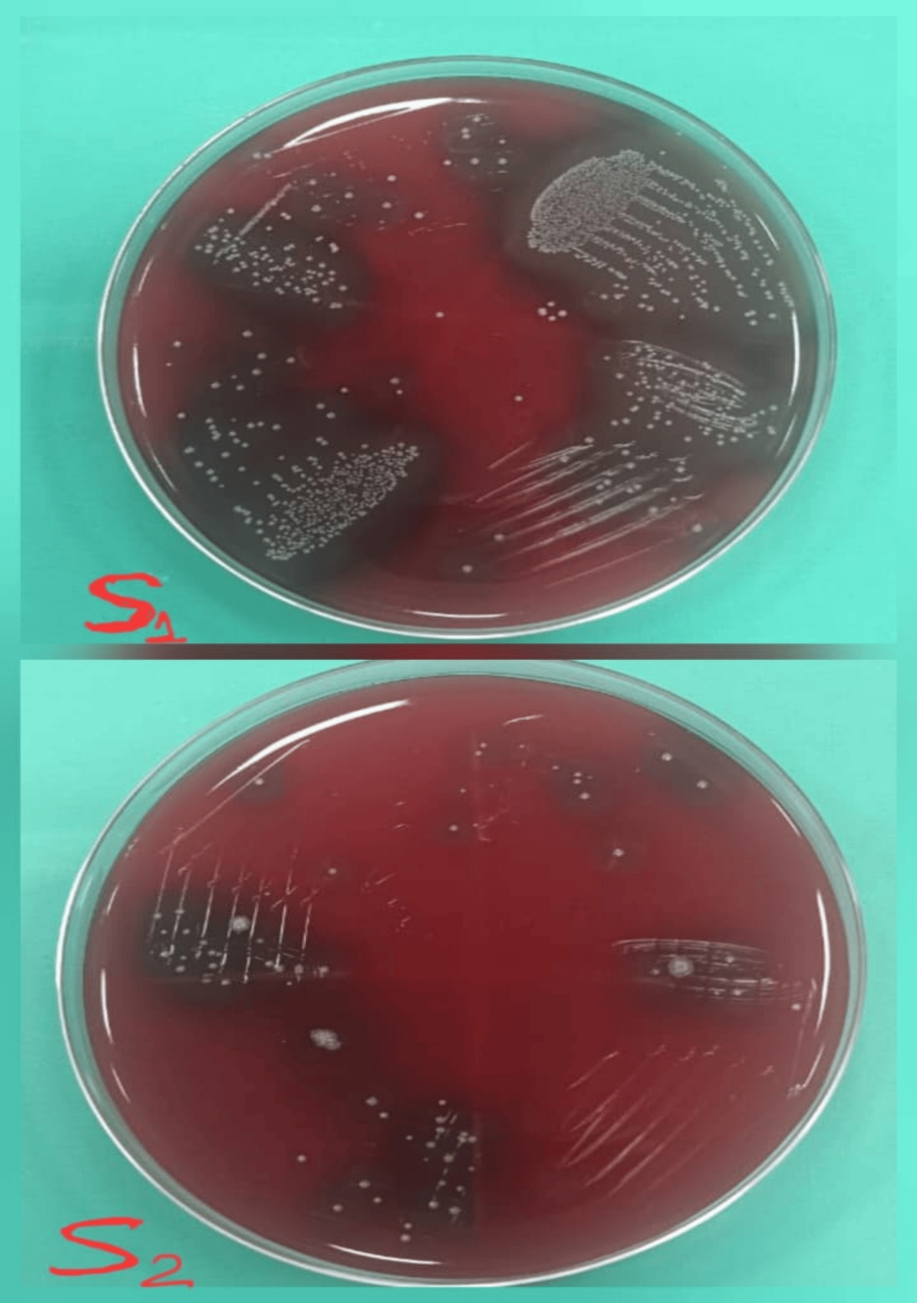
Culture images of the microbial S1 and S2 samples treated with bromelain S1: initial microbial sample; S2: second microbial sample obtained after seven days of medicament application.

**Figure 6:**
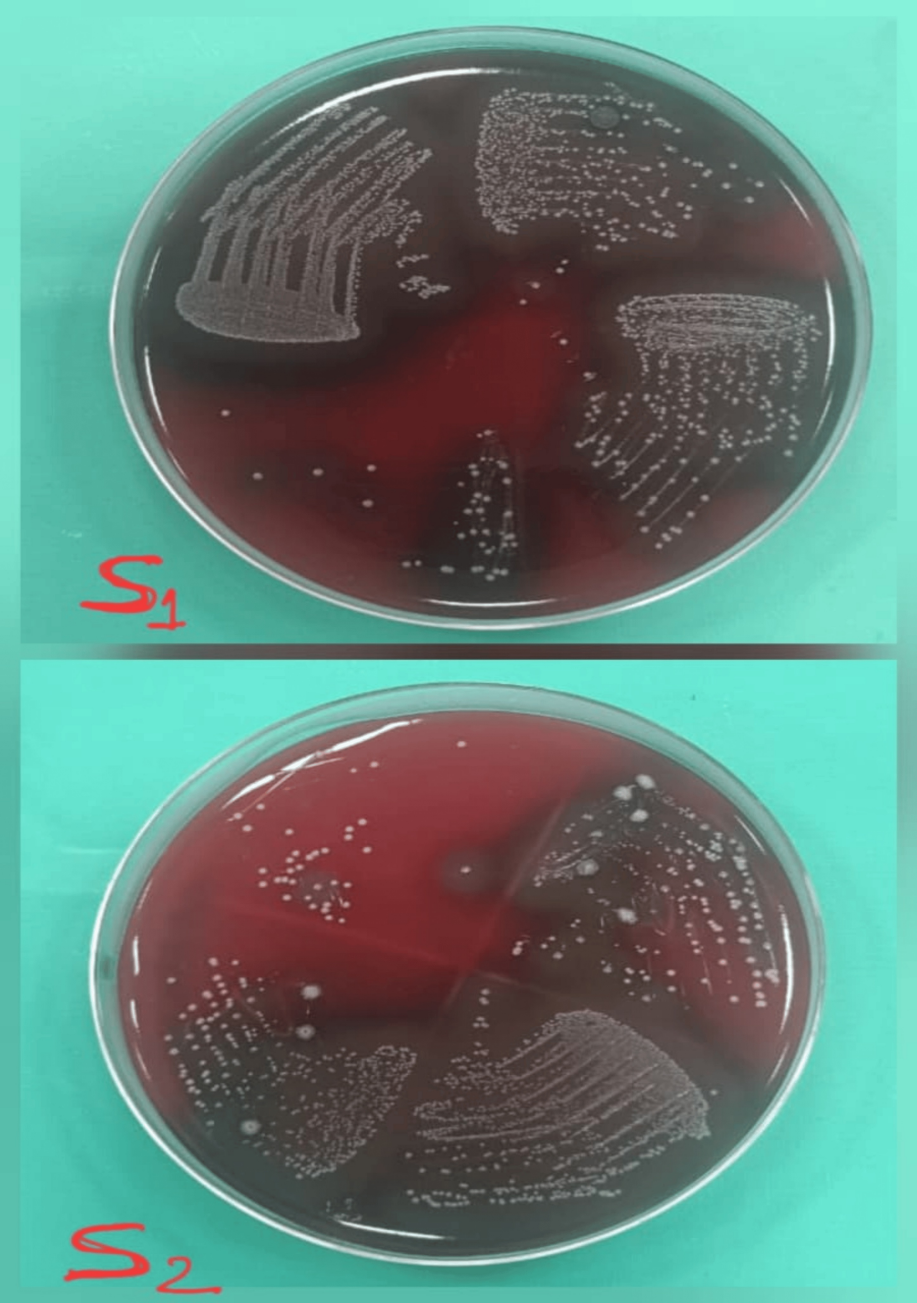
Culture images of the microbial S1 and S2 samples without medicament S1: initial microbial sample; S2: second microbial sample obtained after seven days of medicament application.

Following a medicament application, the canal orifices were closed using sterile cotton pellets and a provisional restoration. Then the samples were incubated at 37°C for another seven days before the second sampling (S2) was performed. To collect the bacterial samples, sterile saline was injected into the canals with a 1 ml insulin syringe. Then, sterile #20 H-files were carefully used to scrape the canal walls to loosen the debris, and paper points were placed inside the canals for 1 minute. The paper points were subsequently immersed in test tubes containing 1 ml of saline and vortexed for 30 seconds, and then were plated onto blood agar (Himedia Laboratories Pvt. Ltd., Mumbai, India) and incubated at 37°C for 48 hours. Finally, CFUs per ml were assessed. Figure 2 shows the culture images for the microbial S1 and S2 samples for Arjuna. Figure 3 shows the culture images for the microbial S1 and S2 samples for calcium hydroxide. Figure 4 shows the culture images for the microbial S1 and S2 samples for TAP. Figure 5 shows the culture images for the microbial S1 and S2 samples for bromelain. Figure 6 shows the culture images for the microbial S1 and S2 samples without medicament.

## Results

**Figure 7:**
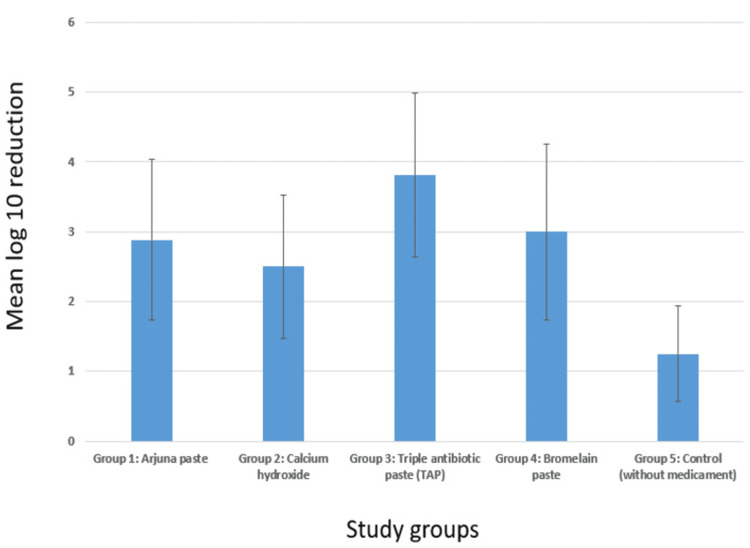
Comparison of mean log₁₀ reduction among the groups Group 1: Arjuna paste - This group shows a mean log₁₀ reduction of approximately 2.9, indicating a significant decrease in the bacterial population. Group 2: Calcium hydroxide - This group shows a mean log₁₀ reduction of approximately 2.5, which is effective but slightly less than the Arjuna paste group. Group 3: Triple antibiotic paste (TAP) - This group shows the highest mean log₁₀ reduction, at approximately 3.8, suggesting it to be the most effective treatment among the five groups in reducing bacterial counts. Group 4: Bromelain paste - This group shows a mean log₁₀ reduction of approximately 3.0, performing slightly better than the Arjuna paste and calcium hydroxide groups. Group 5: Control (without medicament) - This group serves as a baseline, showing a mean log₁₀ reduction of approximately 1.2. This lower value indicates a much smaller reduction in bacterial counts compared to the groups that received a medicament.

**Figure 8:**
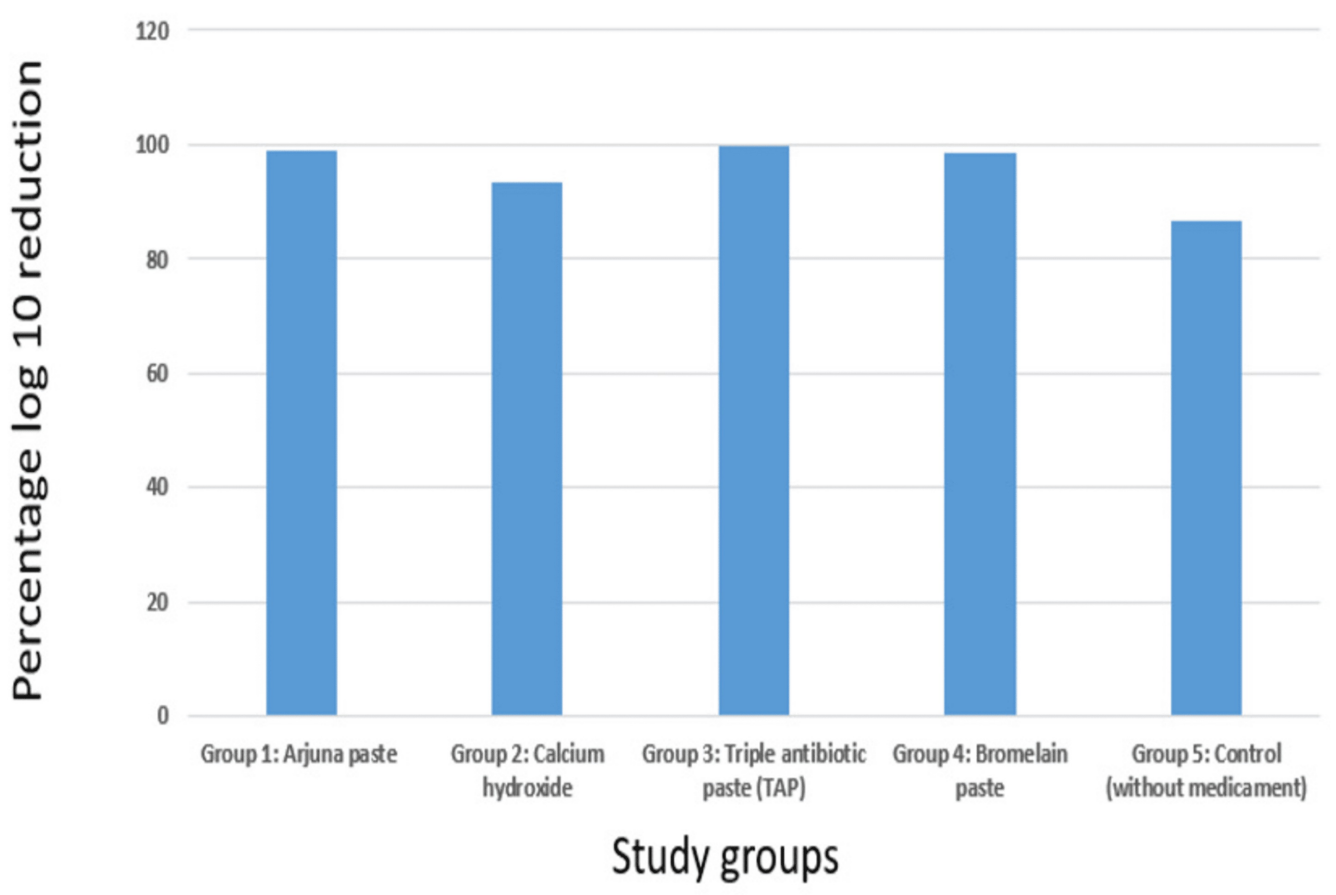
Comparison of percentage log₁₀ reduction among the groups Group 1: Arjuna paste: The bar shows a percentage log₁₀ reduction of approximately 99. Group 2: Calcium hydroxide: The bar shows a percentage log₁₀ reduction of approximately 93. Group 3: Triple antibiotic paste (TAP): The bar shows a percentage log₁₀ reduction of approximately 100. Group 4: Bromelain paste: The bar shows a percentage log₁₀ reduction of approximately 99. Group 5: Control (without medicament): The bar shows a percentage log₁₀ reduction of approximately 87.

Table 1 compares the log₁₀ reduction among five different groups. All investigated medicaments demonstrated significant antimicrobial activity, with percentage reductions in bacterial load ranging from 86.68% to 99.89%. The highest percentage reduction was observed with the TAP (99.89%), followed closely by Arjuna paste (99.02%), bromelain paste (98.57%), and CH (93.27%). The control group, which did not receive any medication, showed the lowest reduction at 86.68%. Figure 7 depicts a comparison of mean log₁₀ reduction among the different groups. Figure 8 depicts the comparison of the percentage log₁₀ reduction among the different groups.

**Table 1:** Comparison of log₁₀ reduction among the groups Statistical analysis was performed using the Kruskal-Wallis test. *Indicates a significant difference at p ≤0.05.

Group	Mean	SD	% Reduction	p-Value
Group 1: Arjuna paste	2.88	1.15	99.02	<0.001*
Group 2: Calcium hydroxide	2.50	1.03	93.27	
Group 3: Triple antibiotic paste (TAP)	3.81	1.17	99.89	
Group 4: Bromelain paste	3.00	1.26	98.57	
Group 5: Control (without medicament)	1.25	0.68	86.68	

A post hoc Bonferroni test was conducted to identify which specific groups differ significantly. A significant difference in log₁₀ reduction between Arjuna paste and the control group (p=0.002) was noted (Table 2). Arjuna paste showed a much higher mean log₁₀ reduction (2.88) compared to the control (1.25). A significant difference was seen between CH and the control group (p=0.032). CH had a higher mean log₁₀ reduction (2.50) than the control. TAP showed a highly significant difference in log₁₀ reduction from the control (p<0.001). TAP had the highest mean log₁₀ reduction (3.81) among all groups. Bromelain paste also demonstrated a significant difference in log₁₀ reduction than the control (p=0.001). Bromelain paste had a higher mean log₁₀ reduction (3.00) than the control.

**Table 2:** Pairwise comparison of log₁₀ reduction among groups Gr1: Arjuna paste; Gr2: calcium hydroxide; Gr3: triple antibiotic paste (TAP); Gr4: bromelain paste; Gr5: control (without medicament). *Indicates a significant difference at p ≤0.05.

Pairwise comparison	Mean difference	SE	p-Value
Gr 1 vs Gr 2	0.38	0.38	1.000
Gr 1 vs Gr 3	-0.93	0.38	0.444
Gr 1 vs Gr 4	-0.12	0.38	1.000
Gr 1 vs Gr 5	1.63	0.38	0.002*
Gr 2 vs Gr 3	-1.31	0.38	0.052
Gr 2 vs Gr 4	-0.50	0.38	1.000
Gr 2 vs Gr 5	1.25	0.38	0.032*
Gr 3 vs Gr 4	0.81	0.38	0.882
Gr 3 vs Gr 5	2.56	0.38	<0.001*
Gr 4 vs Gr 5	1.75	0.38	0.001*

The results indicate that all the medicaments (Arjuna paste, CH, TAP, and bromelain paste) were significantly more effective in reducing the log₁₀ values compared to the control group (without medicament).

Among the investigated medicaments, TAP showed the highest mean log₁₀ reduction, although it did not demonstrate a statistically significant difference compared to the herbal groups. Arjuna was equally effective as bromelain without any significant difference (p=1.000). CH showed the lowest reduction as compared to TAP, bromelain, and Arjuna.

## Discussion

Endodontic infections are polymicrobial in nature, involving complex interactions among diverse bacterial species. Despite the advances in mechanical instrumentation, substantial portions of the RC walls often remain uninstrumented, necessitating the adjunctive use of intracanal irrigants and medicaments to achieve effective disinfection [13]. There is a constant search for ideal intracanal irrigants and medicaments to improve the success rate of the RC procedure. This search has led to the investigation of herbal medication.

In the present study, two new herbal agents, Arjuna and bromelain, were investigated with the commonly used TAP and CH for their effectiveness against EF. According to the present research, TAP demonstrated the strongest antibacterial efficiency against EF with a mean log₁₀ reduction of 3.81 and a 99.89% bacterial reduction. Similarly, Mozayeni et al. found that TAP had significantly higher capabilities than CH in reducing EF, supporting the superiority seen in our findings [14]. However, concerns exist regarding its cytotoxic potential and rare microbial resistance to some TAP components [15].

Despite being a conventional intracanal medication, CH had somewhat less antibacterial activity against E.F. in the current investigation, with a mean log₁₀ decrease of 2.50 and a reduction of 93.27%. This is consistent with the findings by Haapasalo and his team members [16] and Lana and colleagues [17], who noted that EF resists high pH and can penetrate deeply into dentinal tubules, limiting CH’s effect, especially when used for short durations like seven days. Our results agree with this, as the antimicrobial efficacy of CH was statistically lower than that of TAP (p=0.052, close to significance).

With a mean log₁₀ reduction of 3.00 and a 98.57% bacterial reduction, the plant-derived proteolytic enzyme bromelain showed promising antimicrobial activity. Our findings are supported by Arsyada et al., who showed enhanced antimicrobial action when bromelain was combined with CH [18]. Jančič and Gorgieva highlighted bromelain's strong activity against Gram-positive organisms like EF, due to its proteolytic and membrane-disrupting effects [19]. However, Chandwani and her team [11] revealed that bromelain had higher capabilities than the traditional TAP as an intracanal medication against E.F.

Arjuna paste also demonstrated strong antimicrobial efficacy in our study, with a mean log₁₀ reduction of 2.88 and 99.02% bacterial reduction. Although the differences compared to TAP and bromelain were not statistically significant (p=0.444 and p=1.000, respectively), it was significantly more effective than the control (p=0.002) and also CH (p=1.000). These findings are consistent with those of Jain and colleagues [20], who attributed Arjuna's antibacterial effects to its phytochemical constituents like tannins, flavonoids, and triterpenoids. However, Singh et al. [21] reported only moderate antimicrobial performance of Arjuna against EF, when compared to synthetic antibiotics, indicating that differences in extract concentration and preparation may influence efficacy.

Overall, the present study suggests that the natural agents, bromelain and Arjuna, showed encouraging efficacy and outcomes that were equivalent to those of TAP. These findings are in line with studies that have provided favorable results by utilizing various herbal-based intracanal medicaments [22-26].

As an in vitro study, the results may not directly translate to clinical outcomes due to the absence of host factors, polymicrobial infections, and canal complexities. Only EF was used, which, although a commonly isolated pathogen in endodontic failure, does not represent the complete microbial profile. Additionally, only one concentration and a seven-day application period were tested. Further in vivo studies with varied concentrations, longer durations, and combination therapies are necessary to validate these findings.

## Conclusions

In conclusion, the current findings strongly support the earlier research that found TAP to be highly effective against EF. Nonetheless, as natural agents, bromelain and Arjuna showed encouraging efficacy and outcomes that were equivalent to those of TAP, making them desirable prospective substitutes. CH, while historically valuable, demonstrated limited efficacy in this short-term model, underscoring the importance of exposure duration. Future in vivo investigations with standardized delivery protocols and broader microbial targets are essential to establish the clinical applicability of these agents.

## References

[1] Anumula L, Kumar S, Kumar VS (2012). An assessment of antibacterial activity of four endodontic sealers on Enterococcus faecalis by a direct contact test: An in vitro study. ISRN Dent.

[2] Wang Z, Shen Y, Haapasalo M (2012). Effectiveness of endodontic disinfecting solutions against young and old Enterococcus faecalis biofilms in dentin canals. J Endod.

[3] Gaeta C, Marruganti C, Ali IA (2023). The presence of Enterococcus faecalis in saliva as a risk factor for endodontic infection. Front Cell Infect Microbiol.

[4] Atila-Pektaş B, Yurdakul P, Gülmez D, Görduysus O (2013). Antimicrobial effects of root canal medicaments against Enterococcus faecalis and Streptococcus mutans. Int Endod J.

[5] Cehreli ZC, Isbitiren B, Sara S, Erbas G (2011). Regenerative endodontic treatment (revascularization) of immature necrotic molars medicated with calcium hydroxide: A case series. J Endod.

[6] Reynolds K, Johnson JD, Cohenca N (2009). Pulp revascularization of necrotic bilateral bicuspids using a modified novel technique to eliminate potential coronal discolouration: a case report. Int Endod J.

[7] Wigler R, Kaufman AY, Lin S, Steinbock N, Hazan-Molina H, Torneck CD (2013). Revascularization: A treatment for permanent teeth with necrotic pulp and incomplete root development. J Endod.

[8] Maurer HR (2001). Bromelain:  biochemistry,  pharmacology and medical use. Cell Mol Life Sci.

[9] Chaudhari GM, Mahajan RT (2015). Comprehensive study on pharmacognostic, physico and phytochemical evaluation of Terminalia arjuna Roxb. stem bark. J Pharmacogn Phytochem.

[10] Balagopal S, James V, Geethapriya N, Shobana S, Varadarajan S, Jagannathan R, Balaji TM (2023). Herbs and their applications as root canal medicaments. Pharmacological Studies in Natural Oral Care.

[11] Chandwani ND, Gedam UD, Deshmukh R, Dakshindas DM, Shrigiriwar M (2024). Mines of cytokine: A treasure trove in pulpal and periapical diseases. J Conserv Dent Endod.

[12] Rosner BA (2006). Fundamentals of Biostatistics. Fundamentals of biostatistics. Belmont, CA: Thomson-Brooks/Cole.

[13] Wong J, Manoil D, Näsman P, Belibasakis GN, Neelakantan P (2021). Microbiological aspects of root canal infections and disinfection strategies: An update review on the current knowledge and challenges. Front Oral Health.

[14] Mozayeni MA, Haeri A, Dianat O, Jafari AR (2014). Antimicrobial effects of four intracanal medicaments on enterococcus faecalis: An in vitro study. Iran Endod J.

[15] Ruparel NB, Teixeira FB, Ferraz CC, Diogenes A (2012). Direct effect of intracanal medicaments on survival of stem cells of the apical papilla. J Endod.

[16] Haapasalo HK, Sirén EK, Waltimo TM, Ørstavik D, Haapasalo MP (2000). Inactivation of local root canal medicaments by dentine: An in vitro study. Int Endod J.

[17] Lana PE, Scelza MF, Silva LE, Mattos-Guaraldi AL, Hirata Júnior R (2009). Antimicrobial activity of calcium hydroxide pastes on Enterococcus faecalis cultivated in root canal systems. Braz Dent J.

[18] Arsyada IF, Rianti D, Munadziroh E (2018). Antibacterial activity of mixed pineapple peel (Ananas comosus) extract and calcium hydroxide paste against Enterococcus faecalis. Dent J (Maj Ked Gigi).

[19] Jančič U, Gorgieva S (2021). Bromelain and nisin: The natural antimicrobials with high potential in biomedicine. Pharmaceutics.

[20] Jain S, Yadav PP, Gill V, Vasudeva N, Singla N (2009). Terminalia arjuna a sacred medicinal plant: Phytochemical and pharmacological profile. Phytochem Rev.

[21] Amalraj A, Gopi S (2017). Medicinal properties of Terminalia arjuna (Roxb.) Wight &amp; Arn.: A review. J Tradit Complement Med.

[22] Solanki MN, Attur KM, Vachhani KA, Patel NA, Shah MA, Doshi DM (2024). Phytochemicals in vital tooth bleaching: Spectrophotometric evaluation of efficacy with papaya, pineapple, or kiwi extracts and 30% hydrogen peroxide. J Conserv Dent Endod.

[23] Kishan KV, Shah N, Mamatha KV, Sreekumari L, Parikh M (2025). A randomized clinical comparative evaluation of interappointment flare-ups on placing Azadirachta indica, garlic, Triphala, and calcium hydroxide as intracanal medicament, in primary endodontic lesion. J Conserv Dent Endod.

[24] Sharma L, Sinha DJ, Puri N, Dhawan A, Prakash P, Sharif N (2024). Antimicrobial efficacy of 2% chlorhexidine gel, triphala, and Azadirachta indica as intracanal medicaments against Enterococcus faecalis: A randomized clinical trial. J Conserv Dent Endod.

[25] Chandwani ND, Maurya N, Nikhade P, Chandwani J (2022). Comparative evaluation of antimicrobial efficacy of calcium hydroxide, triple antibiotic paste and bromelain against Enterococcus faecalis: An in vitro study. J Conserv Dent.

[26] Jagyasi DR, Chandwani ND, Gunwal MK, Ranka AS (2021). Antimicrobial efficacy of acacia Nilotica (Babul) extract and its effectiveness in disinfecting gutta percha cones - An in vitro study. Indian J Dent Res.

